# Managing Wildlife Disease Under Climate Change

**DOI:** 10.1007/s10393-021-01542-y

**Published:** 2021-08-31

**Authors:** Danielle Buttke, Margaret Wild, Ryan Monello, Gregor Schuurman, Micah Hahn, Kaetlyn Jackson

**Affiliations:** 1 National Park Service Biological Resources Division and Office of Public Health, 1201 Oakridge Drive, Suite 200, Fort Collins, CO 80524 USA; 2 Washington State University College of Veterinary Medicine, Pullman, USA; 3 National Park Service Pacific Island Inventory and Monitoring Network, Hawaii Volcanoes National Park, USA; 4 National Park Service Climate Change Response Program, Fort Collins, USA; 5 University of Alaska Anchorage Institute for Circumpolar Health Studies, Anchorage, USA; 6 Fire Island National Seashore, New York, USA

For many land management agencies, climate change presents an unprecedented circumstance where native species’ ranges are shifting, natural processes are transforming, and previously innocuous stressors can have outsized impacts (Feldmen et al., 2017; Pecl et al., 2017; Byers 2020; Cohen et al., 2020; Rohr & Cohen, 2020). In this space, conservation practices and traditional land-management policy can conflict. This divergence is particularly apparent for agencies such as the United States National Park Service (NPS) that, despite existing in a world changing rapidly under modern human influence, are steeped in long-established policies to maintain natural conditions. How NPS policy and its implementation evolve are critical not only to the agency and its stakeholders, but as an example for other natural resource management organizations with similar preservation missions.

The NPS is mandated to preserve plant and animal species native to park ecosystems and restore natural processes and communities whenever possible (NPS, 2006). Historical management approaches were relatively straightforward: preserve the natural condition defined as ‘the condition that would occur in the absence of human dominance over the landscape’ and manage where threats of resource impairment exist and management is prudent and feasible (NPS, 2006). The climate crisis presents an unparalleled challenge not only in identifying prudent and feasible management under such rapid change but even in determining what species and processes should be considered native or natural. This complexity is evident when dealing with animals and plants and further multiplied for infectious diseases. Complexities of infectious disease ecology, the range of projected environmental changes, and the diversity of pathogens,^1^ vectors, and hosts will make it challenging to both define and protect ‘native’ and ‘natural’ resources, requiring decisions under increasing uncertainty.

Infectious disease dynamics are complex and sensitive to a range of global changes. Climate change can impact geographic or temporal patterns of infectious diseases, but these impacts are often not uniform, predictable, or generalizable across systems (Lafferty, 2009; Cohen et al., 2020). Diseases that involve multiple reservoir or vector species are more likely to be affected by climate change and likely more difficult to model and manage (Rohr et al., 2011). Temperature affects the development of pathogens, hosts, and vectors as well as their habitat and range in multiple ways. For example, higher temperatures can accelerate the development and reproductive rate of ectothermic organisms such as ticks and mosquitoes, resulting in a larger vector population and higher risk of disease transmission, but if it is too hot and dry, this can create inhospitable conditions for vectors and limit their geographic range (Kutz et al., 2009; Liao et al., 2015; van Panhuis et al., 2015; Onyango et al., 2020; Faiman et al., 2017). Higher mean temperatures can also affect temporal disease patterns, for example by causing an earlier questing (i.e., host-searching) season for ticks, or by limiting the hours of the day that are suitable for tick questing due to high heat and low humidity (Brownstein et al., 2005; Tagliapietra et al., 2011; Monaghan et al., 2015). Changes in precipitation due to climate change may impact the occurrence and spread of disease, but the direction and magnitude of impacts are difficult to predict at smaller scales, and will likely vary across the landscape and interact with temperature in unpredictable ways (Harrigan et al., 2018; Hahn et al., 2015; Gardner et al., 2012; Eisen et al., 2010; Shaman et al., 2005; Koenraadt and Harrington, 2008; Herrmann and Gern, 2013; Berger et al., 2014; Cohen et al., 2020). Precipitation-responsive diseases, such as anthrax, hemorrhagic vector-borne diseases, plague, and hantavirus, may see more significant peaks of activity during larger rainfall events, but these may be interspersed with longer periods of inactivity during prolonged and more significant droughts (Engelthaler et al, 1999; Eads & Hoogland, 2017; Walsh et al., 2018). Additionally, variability in temperature and precipitation abnormalities and thermal mismatch, which typically advantages smaller organisms such as parasites and pathogens, can also significantly influence disease dynamics, reducing fitness of cold- or constant-climate adapted species while benefitting species adapted to warmer climates and limiting the geographic range of each (Eads & Hoogland, 2017; Cohen et al., 2020).

For pathogens, hosts, and vectors whose life cycles are limited by temperature extremes, higher mean temperatures will likely cause a pole-ward shift in geographical range (Lafferty, 2009). The ranges of previously separated populations (pathogens, hosts, and/or vectors) may begin to overlap under a changing climate regime, resulting in novel disease assemblages or the expansion of disease risk into new areas. For example, this has been observed as the meningeal worm of white-tailed deer encounters moose and elk populations, or when lifecycle changes occur, as has been observed in muskox lungworms that have expanded their range and accelerated their development under warmer temperatures, with significant clinical impacts on their host (Kutz et al., 2009; Wetzel and Weigl, 1994; Pickles et al., 2013; Feldmen et al., 2017; Kafle et al., 2020). Similarly, as host populations disappear or decrease on the landscape, so too will the pathogens dependent on these species for survival. Although research has shown that a decrease in biodiversity often results in increased disease risk (Civitello et al., 2015; Keesing et al., 2010; Orrock et al., 2011; Johnson et al., 2009; Johnson and Thieltges, 2010; Johnson et al., 2013a,b), climate change could also decrease the prevalence and geographic extent of some diseases.

Historically, disease in free-ranging animals was considered a natural process inherent to the functioning of a park ecosystem, and the NPS did not routinely intervene or attempt to manage disease impacts unless overt public health impacts existed (Sellars, 1997). Disease can be a natural process and is indeed at some level a component of a healthy ecosystem. Traditionally, native disease-causing organisms warranted the same protection as other flora and fauna. However, re-examination of disease origins, in part aided by rapid advances in genetic and other classification techniques, led to the realization that observed diseases were in many cases a result of exotic pathogens or anthropogenic impacts (Meager and Meyer, 1994; NPS, 2002a , b; Wobeser, 2002). This recognition of the nonnative origin and anthropogenic drivers of some major diseases required broadened disease management approaches in free-ranging wildlife.

Today, most if not all ecosystems are impacted by significant external stressors, most notably land-use changes, loss of habitat connectivity, introduced species, changes in species abundance and density, and climate change. These external stressors have already significantly impacted both disease occurrence and wildlife populations such that current disease processes are rarely a completely natural process. Disease is a highly complex process influenced by both infectious and non-infectious factors. Changes in any of the three components of the disease triad – host, agent, or environment – can influence disease outcomes significantly, even if the underlying pathogen itself remains unchanged (Daszak et al., 2001). Diseases are more likely to have significant impacts in a system that has experienced disturbance, whereas non-disturbed systems are more ecologically resilient to disease impacts or introductions (Wobeser, 2002). Active management of diseases should therefore no longer be a last resort but considered a critical tool to ‘mitigate unacceptable impacts from external stressors’ through a harm reduction approach (Colwell et al., 2012; Fisichelli et al., 2016; Stephen et al., 2018). In a wildlife health context, “rather than focusing on obstacles and deficits, [harm reduction] deals with securing critical resources to stay well” (Stephen et al., 2018). Critical harm-reduction strategies for wildlife have included enhancement of wildlife corridors, facilitating genetic exchange across populations, enhancing forage and habitat resources, and reducing impacts of human-wildlife interactions, among others, whether the threats are direct (i.e., vehicular collisions, hunting, or resource competition) or indirect (i.e., reduced reproduction, foraging, and movements due to human presence aversion) (Stephen & Wade 2018; Sleeman et al., 2019; Gallagher, 2020).

Under climate change, ‘natural conditions may be both increasingly difficult to characterize and ineffective as a guide for desired future conditions’ (NPS, 2012). This is particularly true for pathogens and associated diseases. The difficulty and expense in detecting pathogens in free-ranging systems, their rapidly evolving nature, and the lack of historical information on diseases prevents the classification of native versus exotic for many pathogens. As a result, despite NPS policy striving ‘to maintain all the components and processes of naturally evolving park ecosystems, including the natural abundance, diversity, and genetic and ecological integrity of the plant and animal species native to those ecosystems’, a maturation in NPS policy interpretation was required. The decision of how and when to manage an infectious disease in practice is currently based on disease impacts and processes as opposed to disease characteristics (native vs non-native) or drivers (i.e., evolutionary rate, community structure, climate change) (Aguirre et al., 1994, 1995; NPS, 2000, 2006). Whether the pathogen is native or non-native, management is often necessary under anthropogenic change to reduce or prevent local extirpation, genetic compromise, or other impacts to an affected population, as in the case of native epizootic hemorrhagic disease in endangered Florida Key deer. On the other hand, management may be unnecessary when impacts do not compromise population viability, as with the case of climate-driven range expansion of epizootic hemorrhagic disease in white-tailed deer (NPS, 2002a, b; Stallknecht et al., 2015; Zuliani et al., 2015).

Management response to zoonotic diseases creates an additional need for contemporary interpretation. Management Policies (NPS, 2006) directs action in situations where 1) human health and safety are at risk, a disease can be controlled, and human behavior modification, resource use modification, or closures are not effective in protecting human health and safety as determined by public health; 2) disease may pose a threat to adjacent lands; or 3) exotic pathogens that can be managed are threatening native resources. Although this guidance might be interpreted to encourage interventions for diseases that threaten human health even when natural resource impacts result from these interventions, NPS Management Policies section 1.4.3 states:

Congress, recognizing that the enjoyment by future generations of the national parks can be ensured only if the superb quality of park resources and values is left unimpaired, has provided that when there is a conflict between conserving resources and values and providing for enjoyment of them, conservation is to be predominant.

In this context, visitor use of a park may be unacceptable if visitor health can only be preserved through actions that could impair future use or appreciation of native species. In this era of rapid change, more work is needed to proactively identify unacceptable impacts from management actions, as well as visitation itself, to prevent these decisions being made during a crisis.

Climate change is one of many human influences on natural systems. The real risk comes in the rapid and understudied direct impacts, the yet unknown ways in which this and other anthropogenic stressors synergize to impact natural resources, and the limited time that we have to act to prevent irreversible change that threatens natural resources and human civilization alike (IPCC, 2014). Although existing policy can be re-interpreted to accommodate a harm reduction approach, we argue that new or updated Management Policies, Executive Orders, or Congressional Mandates should prioritize and standardize a harm reduction approach and mobilize new and existing resources to better characterize ecosystem ‘health’ and factors that can promote ecological resilience. Managers of national parks and other protected areas are encouraged to take a holistic, preventative approach to infectious disease and evaluate management options from the disease-impact perspective rather than categorization of a specific organism or pathogen. Broader understanding, and therefore data, on the health of our natural resources and pre-determined levels of acceptable impacts and risks are necessary to maximize positive outcomes when management is warranted. Overall, climate change will create more uncertainty and greater susceptibility of populations to stressors such as disease. Taking a harm-reduction approach to conserving natural resources is therefore not only prudent, but our responsibility and best available framework for mitigating impacts.

## Footnotes

1 Infectious disease-causing micro- and macroparasites (pathogens) are not addressed specifically in NPS Management Policies 2006. Here we make the assumption that they are included in the referenced five kingdoms of life and therefore addressed whenever the term “plants and animals” is used in NPS Management Policies (NPS, 2006).
